# Finger tapping at maximal speed evokes crossover fatigability in the other hand

**DOI:** 10.3389/fnhum.2026.1782120

**Published:** 2026-04-07

**Authors:** Caroline Heimhofer, Jenny Imhof, Ingrid Odermatt, Marc Bächinger, Nicole Wenderoth

**Affiliations:** 1 Neural Control of Movement Lab, Department of Health Sciences and Technology, ETH Zurich, Zurich, Switzerland; 2 Neuroscience Center Zurich (ZNZ), University of Zurich, University and Balgrist Hospital Zurich, ETH Zurich, Zurich, Switzerland; 3 School of Psychology, Queen’s University Belfast, Belfast, United Kingdom; 4 Future Health Technologies, Campus for Research Excellence and Technological Enterprise (CREATE), Singapore-ETH Centre, Singapore, Singapore

**Keywords:** crossover fatigability, fatigue, finger tapping, motor fatigability, motor slowing, supraspinal mechanisms

## Abstract

Crossover fatigability (CF) refers to the phenomenon in which fatigability is observed in the non-exercised homologous muscle. As the debate about the existence of CF and its underlying mechanisms is still ongoing and may be protocol-dependent, our study investigated whether CF can be evoked using fast repetitive movements at maximal speed, which have been shown to induce fatigability on the supraspinal level. These rapidly repeating movements induce motor slowing, an involuntary decrease in movement speed. We evoke fatigability through maximal speed finger tapping with one hand and subsequently assessed fast finger tapping performance of the contralateral hand. In two independent cohorts (*n* = 16, *n* = 30), we demonstrated that CF can indeed be induced using a motor slowing paradigm. Our findings show that CF manifests as a reduced initial tapping speed in the other, non-exercised hand and as a reduced “movement reserve.” Notably, even a brief 10 s period of maximal speed finger tapping was sufficient to evoke this CF effect. Based on our current results and on previous findings, we suggest that CF evoked by motor slowing is unlikely to emerge peripherally and most likely reflects supraspinal mechanisms.

## Introduction

Non-local muscle fatigue (NLMF) is a phenomenon that occurs when fatigability is detected in muscles other than the exercised ones (Halperin et al., 2014, 2015; Kennedy et al., 2013). Even though research on NLMF has been performed for over two decades, results are mixed. A recent meta-analysis revealed only limited support for the existence of an NLMF effect (Behm et al., 2021). Multiple determinants have been suggested to influence the occurrence of NLMF (Behm et al., 2021; Halperin et al., 2015). Most prominently, the type of fatiguing protocol is thought to be the most influential factor on the emergence of NLMF, with prolonged or repetitive contractions providing more consistent evidence for NLMF compared to strength or power-based tasks (Behm et al., 2021; Halperin et al., 2015).

The putative origin of NLMF remains unclear, with proposed mechanisms ranging from peripheral to supraspinal levels. One peripheral explanation involves the systemic distribution of performance-diminishing metabolites such as potassium, hydrogen, lactate, and heat shock proteins, which may circulate via the cardiovascular system and impair contractility in muscles not directly involved in the fatiguing task (Behm et al., 2021). In parallel, fatigue-related disturbances in metabolic homeostasis can activate group III and IV muscle afferents, which are known to modulate central motor drive and may therefore contribute to NLMF (Amann, 2012; Amann et al., 2013; Martin and Rattey, 2007). Kennedy et al. (2015), however, found no evidence of group III and IV afferent involvement in crossover effects of fatigability. In addition to peripheral input, central mechanisms such as altered corticomotor excitability have been suggested. Here, the evidence is mixed: some studies report increased excitability in non-fatigued muscles following fatiguing contractions (Aboodarda et al., 2016; Matsuura and Ogata, 2015; Takahashi et al., 2011), whereas others indicate a decrease (Amiri et al., 2022; Bonato et al., 1996). Despite these inconsistencies regarding the directionality of the change in corticomotor excitability, contributions of supraspinal mechanisms to NLMF remain a plausible explanatory factor.

Additional support for the idea that transfer effects of fatigability might be mediated supraspinally comes from research on cross-education, a phenomenon in which unilateral training leads to strength gains in the contralateral, untrained homologous limb (Lee and Carroll, 2007). Although the precise mechanisms remain debated, supraspinal involvement in cross-education is well established (see review Calvert and Carson, 2022). Notably, cross-education is highly specific to the homologous muscle and task (Hortobágyi et al., 1997; Lee and Carroll, 2007). Even though cross-education occurs over a longer timescale than NLMF, both phenomena involve the transfer of a functional change to an effector that was not directly engaged in the preceding activity. Based on these parallels, it is plausible to expect that NLMF, if mediated supraspinally, would likewise exert its greatest effect on the contralateral homologous muscle instead of a non-homologous muscle. Therefore, in the present study, we investigate CF, a specific form of NLMF in which the performance of the unfatigued, contralateral homologous muscle is impaired.

Here, we apply a fatigability paradigm that is primarily mediated by supraspinal mechanisms, called motor slowing (Bächinger et al., 2019; Heimhofer et al., 2024a). Motor slowing refers to the decrease in movement speed that is observed when low-force, fast repetitive movements are executed at maximal speed for a short duration of time. In the present work, we use motor slowing as an operational measure of fatigability, consistent with the definition of fatigability as an objectively measurable decline in performance outcome (Kluger et al., 2013). Unlike other fatigability paradigms that require higher contraction intensities, motor slowing shows only minimal peripheral and spinal involvement (Arias et al., 2015; Kluger et al., 2012; Madinabeitia-Mancebo et al., 2021; Madrid et al., 2016, 2018; Rodrigues et al., 2009). Therefore, the motor slowing paradigm lends itself well to explore the involvement of supraspinal mechanisms in CF.

To our knowledge, it remains unknown whether motor slowing, induced by repetitive maximal finger tapping, can elicit CF. The present study therefore tests whether motor slowing of one hand induces a reduction in maximal tapping speed in the contralateral homologous hand. Using a finger-tapping paradigm in two independent cohorts, we assessed whether maximal tapping with the non-dominant hand (NDH) reduces subsequent maximal tapping speed of the dominant hand (DH), and vice versa. Based on evidence from cross-education and the proposed supraspinal mediation of NLMF, we hypothesized that motor slowing would induce CF, reflected in a reduction in tapping speed in the contralateral homologous hand.

Both experiments include a control condition in which the first hand taps for only 10 s (instead of 30 s) because it has been shown that 10 s of maximal speed tapping evokes substantially less pronounced motor slowing than 30 s of tapping (Bächinger et al., 2019). Including an active control condition is important, as unilateral hand movements can influence contralateral motor activity through mechanisms such as interhemispheric inhibition (Ferbert et al., 1992; Perez and Cohen, 2009). For this reason, active control conditions are generally preferable to passive rest. Nevertheless, 10 s of maximal speed tapping induces a state of minimal, rather than absent, fatigability. Therefore, to investigate the influence of the 10 s tapping condition on the second hand, we perform an additional control analysis in the second experiment by comparing CF to a condition in which the preceding hand remained at rest.

## Methods

### Participants

Sixteen volunteers participated in Experiment 1 (13 female, 27 ± 12.2 years, all right-handed) and 30 in Experiment 2 (17 female, 24 ± 3.7 years, 1 left-handed). Sample size for Experiment 2 was determined *a priori* using G*Power (v3.1), based on the smallest effect size observed in Experiment 1 ( $\eta_{p}^{2}$ = 0.32), with *α* = 0.05 and 1 − *β* = 0.95, yielding a required sample of *N* = 30. Participants self-reported to be free from neurological, neuropsychiatric, or neuromuscular disorders, and not be on psychopharmaceutic medication. Also, they reported no signs of arthritis of the finger joints and no limited motor ability in the fingers. Both studies were conducted in accordance with the Declaration of Helsinki: The studies were approved by the Cantonal Ethics Committee of Zürich (Experiment 1: KEK-ZH 2014-0242, Experiment 2: KEK-ZH 2018-01078) and all volunteers signed informed consent before participating in the study.

### Experimental setup and task

#### Experiment 1

##### Task

The overall motor slowing task that was employed for this experiment consisted of tapping alternately with the index and middle finger of one hand. The participants were instructed to tap as fast as possible with their fingers while keeping the wrist in a stable position and the untapping hand and fingers still. Specifically, they were asked to press the key down and then lift the finger fully off the key for each tap. Because previous work has shown that tapping amplitude does not change during motor slowing (Arias et al., 2015), we did not record the range of motion. The tapping duration was set to either 30 s or 10 s (control), following established motor slowing protocols (Bächinger et al., 2019; Heimhofer et al., 2024b). When starting the task, participants were unaware of the length of the current task.

##### CF paradigm

**Figure 1:**
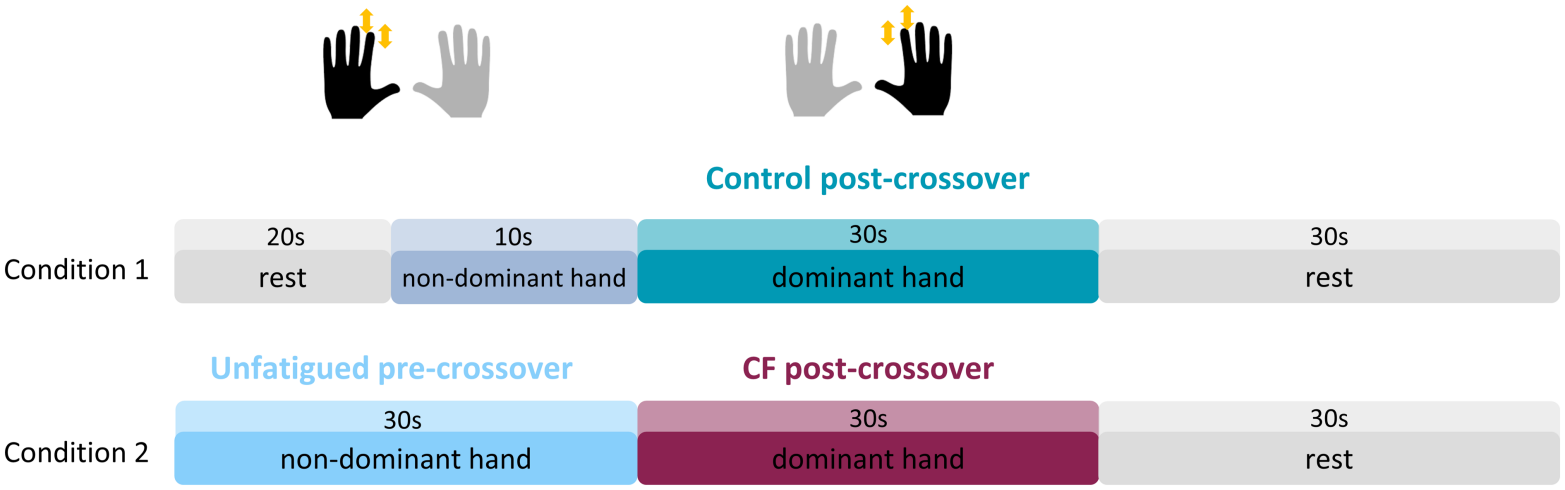
Experimental design for Experiment 1. The experiment consisted of tapping with the NDH for either 10 s, inducing minimal fatigability (blue gray), or 30 s, inducing substantial fatigability in the NDH (light blue, unfatigued pre-crossover condition). This was immediately followed by tapping with the DH. The whole trial in which the NDH tapped for 10 s was referred to as Control (Condition 1), while the whole trials in which the NDH tapped for 30 s was referred to as CF (Condition 2). Tapping behavior in the post-crossover phase was then compared between Control (teal) and CF (merlot).

In this first experiment, we investigated CF from the NDH to the DH, meaning, participants always started tapping with the NDH first, followed immediately by tapping with the DH. The whole experiment consisted of 2 blocks with 20 trials each, i.e., 40 trials in total, split into four different experimental conditions (see further down). Each trial started with a preparatory phase, in which a displayed timer counted backwards for 5 s, followed by finger tapping with the NDH for 10 s or 30 s, followed immediately by finger tapping of the DH for 10 s or 30 s, and then 30 s of rest (Figure 1). The 10 s tapping condition of the NDH was preceded by an additional 20 s break to match the length of the 30 s tapping condition (see Figure 1). The possible combinations of 10 s or 30 s tapping of the NDH and the DH result in four different experimental conditions. Each condition was presented 10 times throughout the experiment, and their order was pseudo-randomized to control for sequence effects. Conditions in which the second, DH tapped for only 10 s were not further analysed for this manuscript.

We refer to tapping with the first hand as the *pre-crossover* condition and tapping with the second hand as the *post-crossover* condition. If the first hand was tapping for 30 s, the condition of the second hand that followed was termed CF condition. Likewise, if the first hand tapped for 10 s, we referred to the condition of the second hand as *Control* condition. We termed this condition as *Control* condition, since we expect only minimal fatigability effects while participants still had to switch from tapping with one hand to tapping with another hand. The 30 s tapping condition of the first hand was termed *Unfatigued (pre-crossover)*, because there was no directly preceding tapping condition, in comparison to *Control* (preceded by 10 s tapping) and to CF (preceded by 30 s tapping).

#### Experiment 2

##### CF paradigm

**Figure 2:**
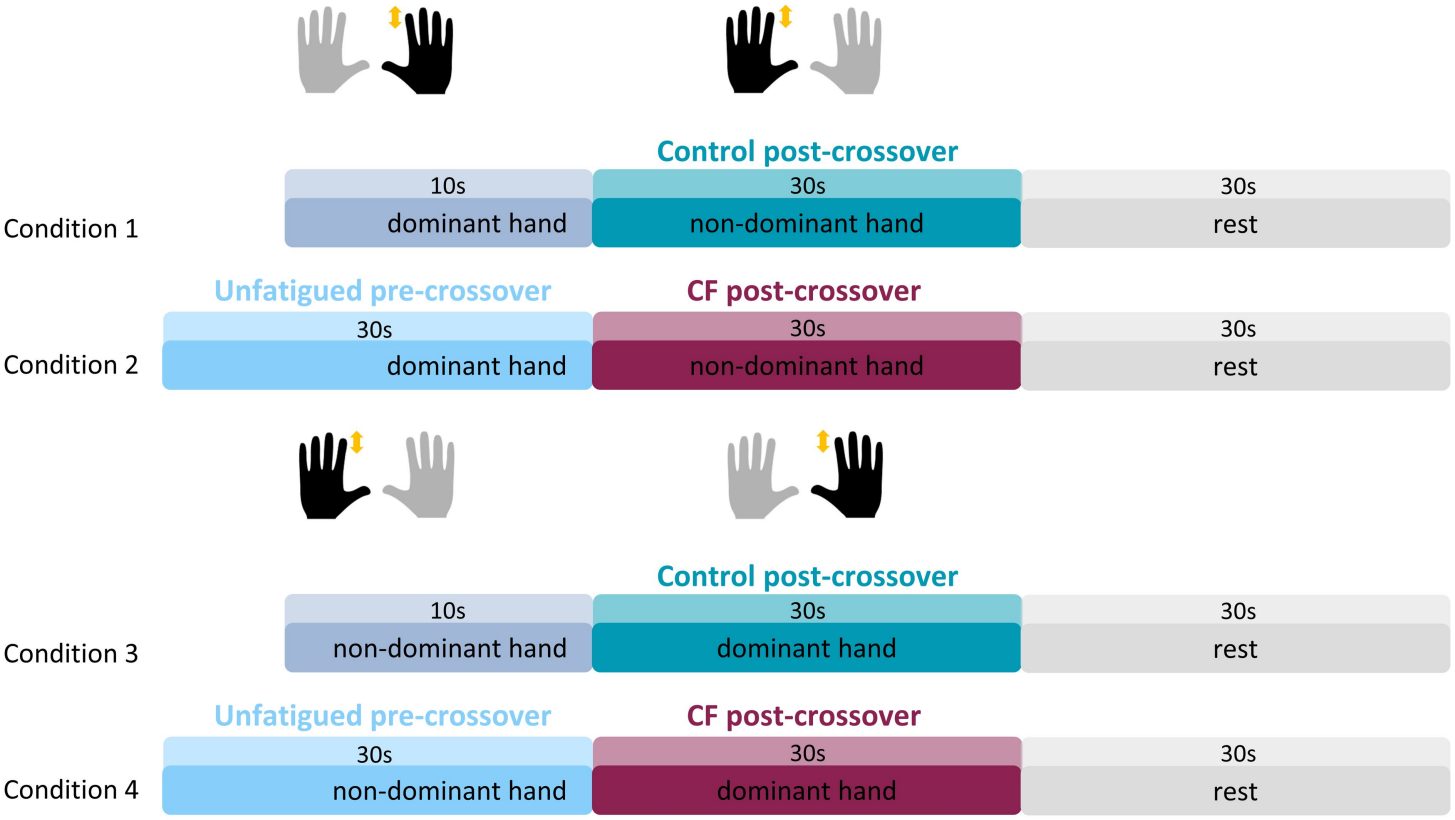
Experimental design for Experiment 2. The experiment consisted of tapping with the NDH or the DH for either 10 s (Conditions 1 and 3) or 30 s (Conditions 2 and 4), immediately followed by tapping with the DH (Conditions 3 and 4) or the NDH (Conditions 1 and 2) for 30s. The light blue and blue-gray represent the pre-crossover condition, in which fatigability is expected to be minimal for 10 s tapping and substantial for 30 s tapping. When compared to the second hand tapping (post-crossover) however, the light blue is referred to as “unfatigued”, because it follows a rest instead of a tapping phase. The teal color indicates the Control post-crossover condition, and the merlot color indicates the CF post-crossover condition.

The task in this second experiment was similar to that of Experiment 1, with one key difference: participants tapped exclusively with the index finger of the designated hand, rather than alternating between the index and middle fingers. The experimental design was adapted from Experiment 1 so that participants began tapping with either the NDH or the DH followed by the opposite hand. This modification allowed us to investigate the CF effect in both directions, i.e., from NDH to DH (as in Experiment 1) and from DH to NDH. Furthermore, the first hand served as an additional control following the rest period. The experiment comprised 3 blocks of 8 trials each, i.e., 24 trials in total, split into four different experimental conditions. Each trial began with a preparatory phase of 5 s, followed by finger tapping with one hand for either 10 s or 30 s, immediately succeeded by tapping with the second hand for 30 s, and concluding with a 30 s rest period. Thus, the experiment included four conditions, differing in whether tapping started with the DH or NDH and whether the initial tapping phase lasted 10 s or 30 s (Figure 2, Conditions 1–4). Each condition was repeated 10 times, and the order of conditions was pseudorandomized.

#### Setup

The experiments were controlled with MATLAB (MathWorks, Experiment 1: version R2015, Experiment 2: version 2020b) and the instructions were displayed with Psychtoolbox (version 3; Brainard, 1997). The finger taps were recorded with a standard computer keyboard for Experiment 1 and with a bimanual response box for Experiment 2. In Experiment 1, a visual cue informed the participants to change from the first to the second hand. Experiment 2 additionally had an auditory cue complementing the visual cue.

### Data analyses

#### Processing

For both experiments, the tapping data were processed in MATLAB (R2020b, MathWorks). Key presses performed with incorrect fingers were removed from the analysis. In the first experiment, in which participants tapped alternately with two fingers, we additionally excluded double presses on the same key. Tapping frequency was calculated and split into 10 s time bins. For statistical analysis, the individual trials were aggregated per participant for each condition. For the 30 s tapping conditions, we additionally calculated the percentage decrease in movement speed from the first to the last time bin and used this decrease as an index for motor slowing (Heimhofer et al., 2024b).

### Statistical analysis

Statistical analysis was performed with R (version 2024.02). To detect and remove outliers in the data, the robustbase package (Maechler et al., 2023) was used. Linear mixed effects models (LMEM) were calculated with the lme4 package (Bates et al., 2023) and type III ANOVA tables were calculated with the lmerTest package (Kuznetsova et al., 2020). In all models, a random intercept for participant was added to account for individual speed offsets. Further, the effectsize package (Ben-Shachar et al., 2023) was used to calculate partial eta squared ( $\eta_{p}^{2}$ ) and the emmeans package (Lenth et al., 2024) was used for post-hoc comparisons with Bonferroni correction.

#### Experiment 1

We ran two main analyses: First, to investigate CF and determine whether tapping with the NDH induced a CF effect on the immediately following DH, we tested whether the tapping speed of the first time bins of the post-crossover conditions differed between Control and CF. This was achieved by a LMEM on tapping frequency in dependence of the factor *Condition* (CF post-crossover, Control post-crossover).

Second, we investigated whether tapping of the NDH influenced the extent of motor slowing, i.e., the percentage decrease in movement speed, of the second DH. For this, we fitted a LMEM on the percentage decrease in dependence of *Condition* (CF post-crossover, Control post-crossover).

Additionally, we performed two control analyses. First, to make sure that the initial pre-crossover conditions were similar, we checked whether the bin of the 10 s tapping condition (0–10 s) and the first bin of the 30 s tapping condition (0–10 s) did not significantly differ from each other by fitting a LMEM on tapping frequency in dependence of *Condition* (10 s, 30 s). Second, to evaluate whether motor slowing is present in all 30 s tapping conditions, we checked whether these conditions showed a statistically significant decrease in movement speed. We tested the 30 s tapping *Unfatigued* pre-crossover condition, *Control* post-crossover, and *CF* post-crossover separately by fitting an LMEM on tapping frequency as a function of *Time* (0–10 s, 10–20 s, 20–30 s bin). Motor slowing was defined to be present if the models revealed a significant main effect of *Time*.

#### Experiment 2

In Experiment 1, the CF effect could only be investigated from the NDH to the DH. In Experiment 2, we could investigate the CF effect also in the other direction, that is, from DH to NDH. Additionally, we now had a pre-crossover condition of each hand, i.e., tapping was performed in an unfatigued state since it followed rest. This unfatigued tapping condition was included in the analyses, when informative.

We quantified the magnitude of the CF effect by calculating the percentage decrease in tapping speed from the average tapping speed of the first time bin from the pre-crossover condition to the tapping speed of the first time bin from the post-crossover conditions of the same hand:


$$\mathrm{CF}\;\text{effect}(\%)=100-\frac{{\text{Post}}_{\mathrm{bin}1}\times 100}{{\mathrm{Pre}}_{\text{average},\mathrm{bin}1}}$$

We calculated this effect separately for CF and *Control*, as well as for each hand. To then determine whether this magnitude of the CF effect differed between CF and *Control*, and whether the effect depended on which hand was used first, we fitted a LMEM on the CF effect in dependence of *Condition* (CF, Control), *Hand* (DH, NDH), and their interaction.

As in Experiment 1, we were interested in defining whether the first hand influenced the tapping behavior of the second hand. Additionally, we wanted to know whether the amount of motor slowing differs between tapping in an unfatigued state (i.e., following rest) versus tapping in the post-crossover conditions (i.e., following 10 s or 30 s tapping of the other hand). Therefore, we also included the unfatigued 30 s pre-crossover condition in this analysis. Hence, we fitted a LMEM on motor slowing (% decrease) of the 30 s tapping conditions in dependence of *Condition* (Unfatigued pre-crossover, Control post-crossover, CF post-crossover), *Hand* (DH, NDH), and the interaction of these two factors.

Finally, we also performed the same control analyses that are listed in Experiment 1 with an additional factor of *Hand* (DH, NDH) and an interaction effect of *Condition* × *Hand*.

## Results

### Experiment 1

**Figure 3:**
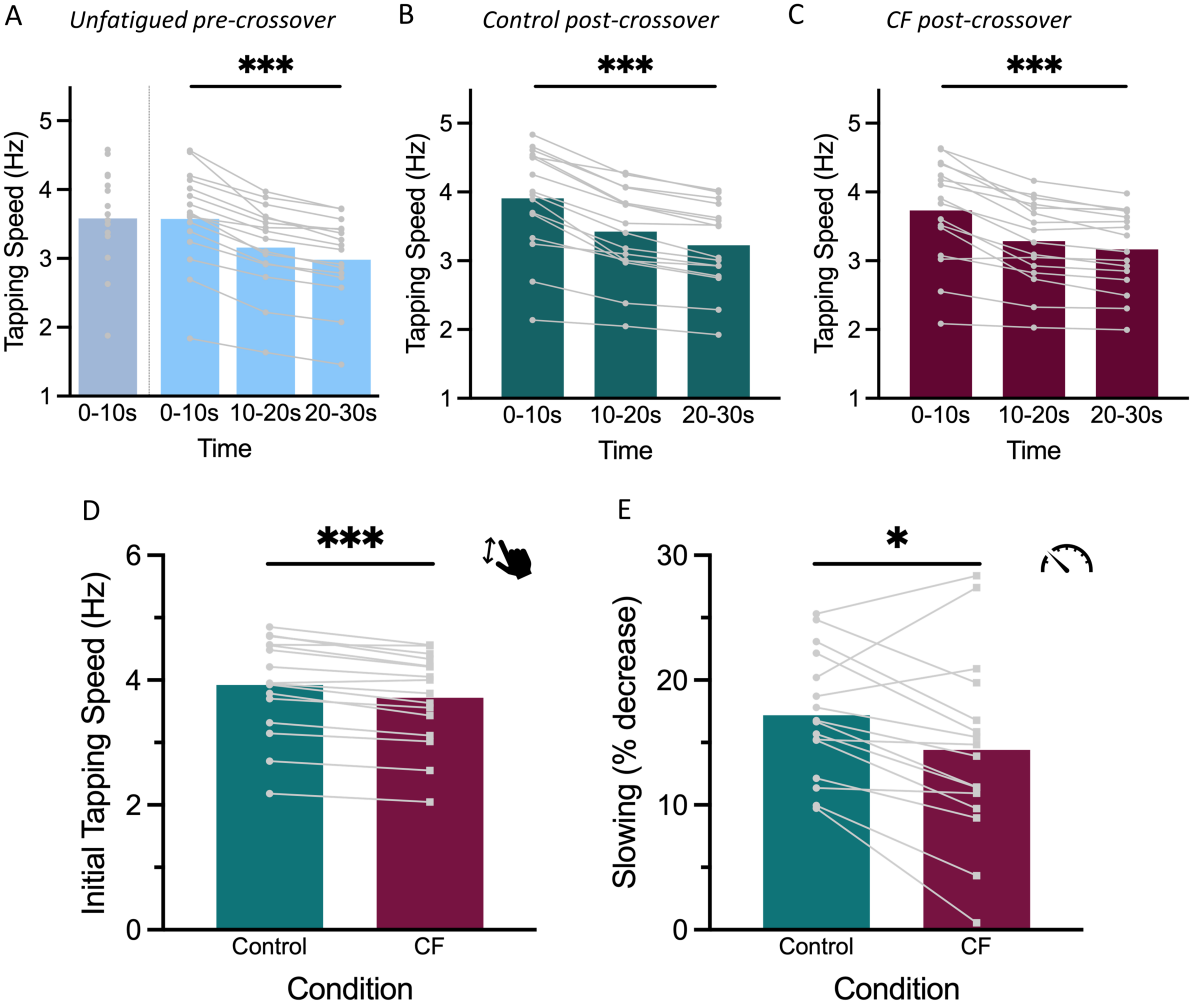
Results of Experiment 1. Colors indicate experimental conditions: blue for pre-crossover tapping with blue-gray for the 10 s tapping condition and light blue for the 30 s tapping condition, teal for post-crossover Control tapping, and merlot for post-crossover CF tapping. Light gray lines represent individual subject averages. The data shown reflect tapping with the DH. Statistical significance is marked with asterisks (^*^p < 0.05, ^**^p < 0.01, and ^***^p < 0.001). **(A–C)** Tapping speed over time, illustrating motor slowing in each condition. **(A)** Unfatigued pre-crossover. **(B)** Control post-crossover. **(C)** CF post-crossover. **(D)** Initial tapping speed comparison between Control and CF. **(E)** Extent of motor slowing, shown as the percentage decrease in tapping speed from the first 10 s to the last 10 s, compared between Control post-crossover and CF post-crossover tapping.

We first inspected whether motor slowing was present in the different conditions. As expected, we observed significant motor slowing not only in the 30 s tapping pre-crossover condition [*F* (2, 32) = 97, *p* < 0.001, $\eta_{p}^{2}$ = 0.86, Figure 3A], but also in both the *Control* post-crossover [*F* (2, 32.002) = 108.960, *p* < 0.001, $\eta_{p}^{2}$ = 0.87, Figure 3B] and the *CF* post-crossover conditions [*F* (2, 32) = 48.565, *p* < 0.001, $\eta_{p}^{2}$ = 0.75, Figure 3C].

Next, as our primary outcome, we investigated whether a CF effect could be detected in the initial post-crossover tapping speed of the second hand. During the first 10 s, tapping speed was significantly lower in the *CF* condition (3.7 Hz ± 0.7 Hz) than the *Control* post-crossover condition (3.9 Hz ± 0.8 Hz), as revealed by a main effect of *Condition* [*F* (1, 48) = 75.170, *p* < 0.001, $\eta_{p}^{2}$ = 0.61, Figure 3D].

As our secondary outcome, we also investigated whether the amount of motor slowing (i.e., the percentage decrease in movement speed over the 30 s tapping period) differed between conditions and found that it was significantly higher in the *Control* post-crossover (17.2% ± 5.0%) than in the *CF* post-crossover condition [14.4% ± 7.4%, main effect of *Condition*: *F* (1, 16) = 7.383, *p* = 0.015, $\eta_{p}^{2}$ = 0.32, Figure 3E].

Additionally, we checked that the observed effects were not due to differences in the initial tapping speed of the 10 s and 30 s pre-crossover conditions and found only insignificant differences [*F* (1, 16) = 0.301, *p* = 0.5912].

In summary, we found reduced tapping speed and less motor slowing in the DH, if it was preceded by NDH tapping at maximal speed for 30 s compared to 10 s, which induced fatigability in from of significant motor slowing in the NDH.

### Experiment 2

In this second experiment, the DH and the NDH were both used as the first hand (*pre-crossover*), as well as the second hand (*post-crossover*) which allowed us to investigate the CF effect from DH to NDH, as well as from NDH to DH.

**Figure 4:**
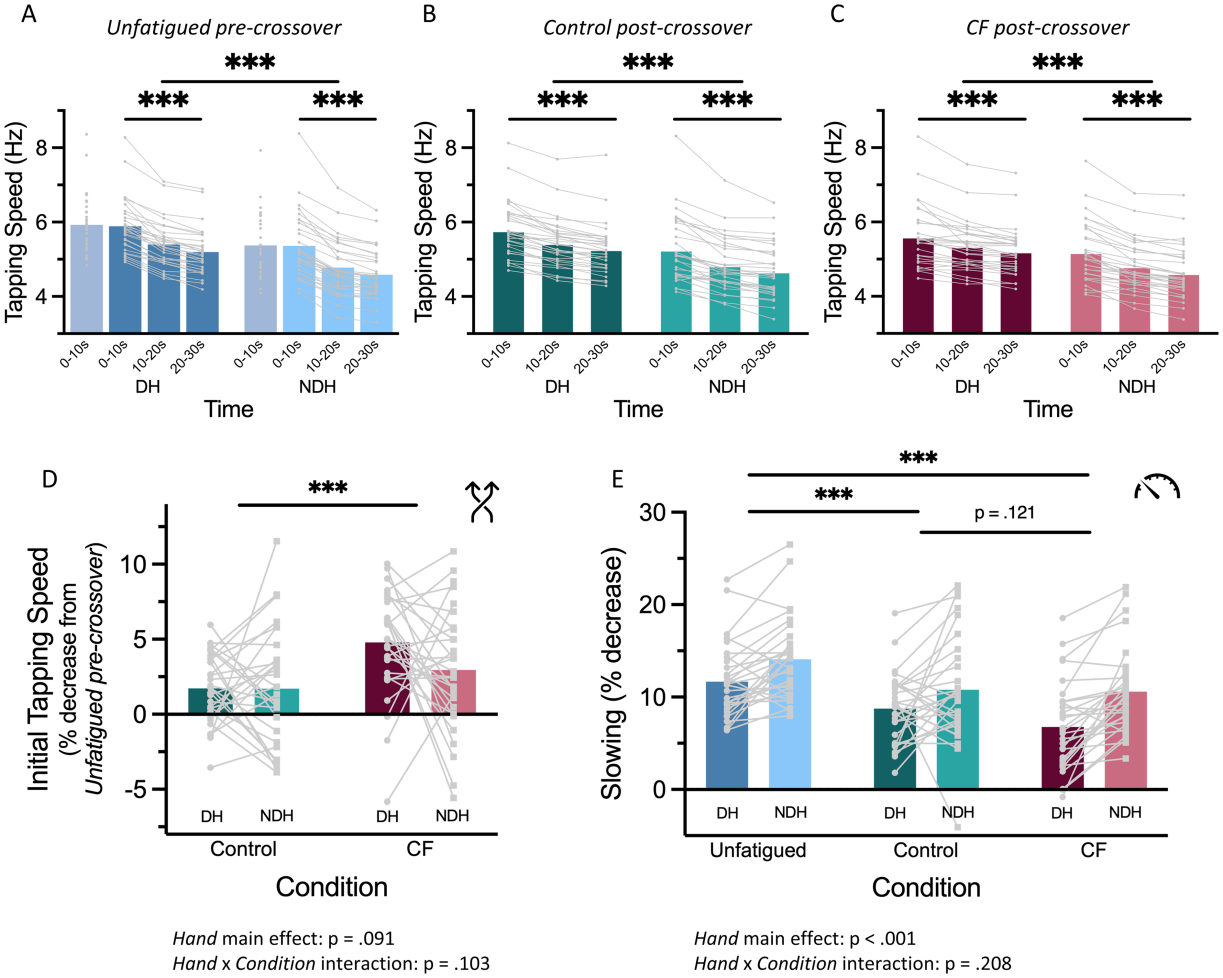
Results of Experiment 2. **(A–C)** Motor slowing depicted as a decrease in tapping speed over time for Pre-crossover (blue, **A**), Control post-crossover (teal, **B**) and CF post-crossover (merlot, **C**). The lighter colors indicate the NDH and the darker colors the DH. The blue-gray bars in **(A)** represent the 10 s tapping condition. **(D)** Magnitude of the CF effect, calculated as percentage decrease of the initial tapping speed from the unfatigued pre-crossover condition of the same hand, shown separated by condition and hand for Control post-crossover (teal) and CF post-crossover (merlot). Darker colors mark DH and lighter the NDH. **(E)** Differences in the extent of motor slowing, i.e., percentage decrease in tapping speed from first 10 s to last 10 s of Pre (blue), Control (teal), CF (merlot), separated by condition and hand with darker colors marking DH and lighter the NDH. Bars without clear group allocation indicate main effects from the LMEMs. Significances are indicated with asterisks: ^*^p < 0.05, ^**^p < 0.01, and ^***^p < 0.001.

We first established that motor slowing was present in each of the experimental conditions requiring 30 s of tapping (Figures 4A–C) and observed a significant decrease in tapping speed over time [main effect of *Time: F* (2, 150) ≥ 62.833, *p* < 0.001, $\eta_{p}^{2}$ ≥ 0.46]. We also found that the DH tapped generally faster than the NDH [main effect of *Hand: F* (2, 150) ≥ 212.802, p < 0.001, $\eta_{p}^{2}$ ≥ 0.59] but found no evidence that the time course of motor slowing differs between hands [no *Time* × *Hand* interaction effect for any condition, all *F* (2, 150) ≤ 1.8614, *p* ≥ 0.159].

Next, as our primary outcome, we investigated whether a CF effect can be detected for the initial tapping speed. In Experiment 2, each hand was tapping in three conditions: (i) after a 30 s break (*Unfatigued pre-crossover*), (ii) immediately after 10 s tapping with the other hand (*Control post-crossover*), and (iii) immediately after fatiguing 30 s tapping with the other hand (*CF post-crossover*). This allows us to quantify the magnitude of the CF effect, estimated via the percentage decrease in initial tapping speed and the extent of motor slowing of *CF post-crossover* and *Control post-crossover* relative to the *Unfatigued pre-crossover* condition of the same hand (Figure 4C).

We replicated the results of Experiment 1 and found that, averaged across hands, the reduction in initial tapping speed was significantly larger in the *CF post-crossover* condition (3.9% ± 3.9%) compared to the *Control* condition [1.7% ± 3.2%, main effect of *Condition*: *F* (1, 90) = 15.409, *p* < 0.001, $\eta_{p}^{2}$ = 0.15]. Interestingly, we also found a reduction in initial tapping speed for *Control post-crossover* relative to *Unfatigued pre-crossover* condition (intercept of the LMEM: *b* = 1.734, SE = 0.630, *t* = 2.751, *p* < 0.01). This indicates that 10 s of tapping with the other hand already induces a CF effect as reduced initial tapping speed, although the effect is substantially smaller than that observed after 30 s of tapping with the other hand (Figure 4C).

In *CF post-crossover*, even though the reduction of initial tapping speed was qualitatively larger in the DH 4.8% (±3.5%) than in the NDH hand 3.0% (±4.1%), we found no significant main effect of *Hand* [*F* (1, 90) = 2.914, *p* = 0.091] and no significant *Condition* × *Hand* interaction (*F* (1, 90) = 2.710, *p* = 0.103).

Finally, as our secondary outcome, we compared the extent of motor slowing (i.e., the percentage decrease from time bin 1 to time bin 3) between the different 30 s tapping conditions (*Unfatigued pre-crossover*, *Control post-crossover*, and CF *post-crossover*). We found that the extent of motor slowing depended on *Condition* [*F* (2, 150) = 34.760, *p* < 0.001, $\eta_{p}^{2}$ = 0.32, Figure 4E]: with 12.9% (±4.4%), *Unfatigued pre-crossover* had the largest decrease in movement speed, followed by *Control post-crossover* with 9.8% (±5.0%) and then *CF post-crossover* with 8.7% (±5.1%). While *post-hoc* tests revealed that *Unfatigued pre-crossover* had a significantly larger decrease in movement speed compared to *Control post-crossover* [*t* (155) = 7.904, *p* < 0.001] and to *CF post-crossover* [*t* (155) = 5.837, *p* < 0.001], we found no significant difference between *CF post-crossover* and *Control post-crossover* [*t* (155) = 2.067, *p* = 0.121]. The extent of motor slowing was larger for the non-dominant than the DH [main effect of Hand: *F* (1, 150) = 41.994, *p* < 0.001, $\eta_{p}^{2}$ = 0.22], but there was no significant interaction effect of *Hand* × *Condition* of the LMEM [*F* (2, 150) = 1.588, *p* = 0.208, $\eta_{p}^{2}$ = 0.02].

As with Experiment 1, we also ensured that the observed effects were not based on differences in initial tapping speed of the pre-crossover conditions. We compared the first 10 s of the 30 s tapping condition and the 10 s tapping condition. This analysis again revealed no significant difference in tapping speed between the two conditions [*F* (1, 90) = 0.292, *p* = 0.591]. Even though there was a significant main effect of *Hand* [*F* (1, 90) = 133.107, *p* < 0.001, $\eta_{p}^{2}$ = 0.6] indicating that DH tapped significantly faster than NDH, there was no significant *Condition* × *Hand interaction* [*F* (1, 90) = 0.067, *p* = 0.795].

## Discussion

In the present study, we show in two independent cohorts that maximal speed finger tapping which induces motor slowing (i.e., the involuntary reduction in tapping speed) evokes CF in the homologous finger of the other hand. We used maximal speed finger tapping for 10 s versus 30 s to induce different levels of fatigability as indicated by stronger “motor slowing” the longer participants tap. We tested how motor slowing of the first hand affects tapping speed of the second hand when tapping started immediately after switching hands. CF manifested as a reduced initial tapping speed in the second hand, irrespective of whether the second hand was the dominant or the non-dominant hand. However, this magnitude of the CF effect depended on the duration of pre-fatiguing tapping: CF was smaller after 10 s than after 30 s of tapping. Additionally, we found that CF occurred in parallel with reduced motor slowing (i.e., a smaller decrease in tapping speed) in the second hand.

Across both experiments, we observed that initial tapping speed was reduced following unilateral fatiguing activity (Figures 3D, 4D). Specifically, tapping speed in the CF condition was consistently lower than in the control condition. These findings demonstrate that 30 s of maximal-speed finger tapping with one hand manifests as reduced motor output in the non-exercised hand, indicating a crossover effect of fatigability. Interestingly, we observed that even our control condition, in which the first hand tapped for only 10 s, had a measurable effect on the outcome of the second hand. This is reflected in two key findings. First, the magnitude of the CF effect quantified by a reduction in initial tapping speed was bigger in the control post-crossover condition compared to the unfatigued pre-crossover tapping with the same hand (Figure 4D). Second, the amount of motor slowing in the unfatigued pre-crossover condition was greater than in the control post-crossover condition (Figure 4E). Thus, both initial tapping speed and the extent of motor slowing were reduced when the hand had been active shortly before, even if only for a brief 10 s period. Note that, while we refer to the 10 s maximal speed tapping condition as our ‘control’, this does not imply that motor slowing was completely absent during such a short period. In fact, previous work by Bächinger et al. (2019) has shown that maximal tapping speed begins to decline immediately after movement onset. Consistent with this, when plotting the 10 s tapping data in three time bins, we also observed a clear decrease in tapping speed over time (Supplementary Figure 1). In summary, 10 s of motor slowing already exerts a measurable effect on contralateral fatigability, speaking to the sensitivity of our measurements. This effect becomes stronger when the initial hand experiences more substantial motor slowing, as seen in the 30 s condition. While CF, or more generally NLMF effects, have not been consistently demonstrated in prior work (for review see Behm et al., 2021), our findings across two independent cohorts provide clear evidence that fast finger tapping which evokes motor slowing reliably induces CF under controlled conditions.

In line with the observation of CF in initial tapping speed, both experiments also revealed effects on the amount of motor slowing. In Experiment 1, where only the DH was tested in the post-crossover conditions, motor slowing in the CF condition was clearly reduced compared to Control post-crossover tapping (Figure 3E). In Experiment 2, which included both the dominant and NDH, a significant main effect of condition on motor slowing was observed (Figure 4E), however, this effect was mainly driven by the strongest slowing effect during the unfatigued pre-crossover condition, the direct comparison between the CF and the Control post-crossover conditions did not reach significance. Note however, that the latter comparison averages across hands while Figure 4E suggests that motor slowing differences between the CF and Control post-crossover conditions might be stronger for the DH than the NDH hand. Overall, the findings demonstrate a consistent pattern of CF across experiments, which is evidenced by reduced initial tapping speed and diminished motor slowing, although the extent of motor slowing may partly depend on hand dominance.

Why do we observe a tendency toward reduced motor slowing in the second hand following prior exertion of the contralateral hand? Studies investigating CF, or more generally NLMF, have typically assessed the crossover effect by comparing post-fatigue performance to a rested baseline (e.g., Behm et al., 2021; Halperin et al., 2015), with outcome measures such as peak force or endurance time. This approach aligns with our comparison of initial tapping speed of the post-crossover conditions, which showed reduced tapping compared to the unfatigued pre-crossover condition. However, by also quantifying the percentage decrease in tapping speed over time within the second hand, our study treats fatigability as a dynamic, evolving process, inspected relative to the starting speed. This additional perspective suggests that starting from a lower initial tapping speed post-crossover may lead to the system reaching a steady-state level of motor output more quickly, indicated by a smaller “tapping speed reserve”. Our prior work showing that movement speed during repetitive tapping rapidly declines and then stabilizes (Bächinger et al., 2019) supports this interpretation. Thus, the CF effect appears to reduce the initial tapping speed of the second hand, thereby requiring a smaller subsequent decrease to reach the final steady-state level, which may explain why motor slowing in the post-crossover tapping period is attenuated.

In line with previous findings (Ashendorf et al., 2009), tapping speed was higher in the DH compared to the NDH. Additionally, the amount of motor slowing was lower in the dominant than the NDH. Earlier studies on asymmetrical transfer in motor learning and strength tasks report stronger transfer from the DH to the NDH (Farthing et al., 2005; Kumar and Mandal, 2005). Our data suggests that crossover fatigability might be more pronounced in the DH than in the NDH, however, this qualitative observation was not supported by the statistics which did not reveal any *Condition* × *Hand* interactions.

What remains unclear are the underlying processes that give rise to the CF effect. One proposed mechanism involves the systemic distribution of performance-diminishing metabolites, which could potentially impair contractility in unfatigued muscles. In this context, the role of group III and IV muscle afferents has been discussed, as the accumulation of such metabolites may influence spinal or cortical excitability through these afferents (Broxterman et al., 2017). However, for our motor slowing CF paradigm, the involvement of metabolites appears unlikely. First, motor slowing has been shown to not be strongly related to the accumulation of performance-diminishing metabolites that activate group III and IV afferents (Madinabeitia-Mancebo et al., 2021), making it improbable that such processes underlie the observed CF effect. Second, the relatively short duration of our tapping protocol of 30 s, or even only 10 s in the control condition, is likely insufficient for metabolites to accumulate and be transported to the contralateral homologous muscle. Together, these considerations suggest that these peripheral, metabolite-related mechanisms may not be the primary drivers of CF in this context.

Instead, we propose that CF induced through motor slowing may be mediated primarily via supraspinal mechanisms. The contribution of central factors to fatigability in general is known to vary depending on the nature of the fatiguing task (Taylor and Gandevia, 2008), and motor slowing itself has been shown to originate from supraspinal processes, including disrupted surround inhibition and altered intracortical inhibition (Bächinger et al., 2019; Heimhofer et al., 2024a). Supporting this idea, even simple unimanual movements have been found to modulate excitability in the ipsilateral motor cortex (Carson et al., 2004; Liepert et al., 2001; Sohn et al., 2003), with more pronounced and task dependent changes emerging under fatiguing conditions. Acute contractions have been shown to alter corticomotor excitability in the contralateral homologous muscle (Aboodarda et al., 2016; Amiri et al., 2022), while longer-term unilateral training induces changes in excitability and inhibition in the ipsilateral hemisphere (Colomer-Poveda et al., 2019). These findings further suggest that changes in excitability and inhibition within the motor cortex contralateral to the fatiguing hand could, possibly via interhemispheric pathways, influence the state of the ipsilateral hemisphere and manifest behaviourally as CF. Notably, the direction and magnitude of such acute neural changes appear to be protocol dependent, which may help explain the variability observed across studies investigating CF.

A key limitation of the present study is that we did not include direct neurophysiological measures to assess cortical excitability or inhibition. Although the motor slowing paradigm is thought to be primarily supraspinally mediated, our suggestions regarding underlying mechanisms are based solely on behavioral outcomes. Therefore, the proposed supraspinal contribution to CF remains speculative. Future studies should directly examine the neurophysiological correlates of CF induced by motor slowing.

While we are aware that we did not actively control that the non-tapping hand was resting, the experimenter watched out for and did not observe any overt movements. Therefore, it is unlikely that unintended movements of the resting hand contributed to the observed results.

Taken together, our findings establish that evoking motor slowing is a reproducible approach for eliciting CF, even though the existence and consistency of this phenomenon have previously been debated. The ability to evoke CF through a well-controlled, short-duration motor task provides a practical and reliable way to study supraspinal contributions to CF and, more broadly, NLMF. CF effects observed with motor slowing may be mediated supraspinally, which is in line with previous studies considering motor slowing to reflect mainly central mechanisms. By using motor slowing as an experimental task, future studies can systematically probe the neural substrates of interlimb interactions, offering the opportunity to better understand the central contributions to fatigability, independent of peripheral or metabolic confounds.

## Data Availability

The original contributions presented in the study are publicly available. This data can be found here: https://doi.org/10.3929/ethz-c-000797084.

## Supplementary material

Supplementary Figure 1 10 s tapping condition of the first hand of experiment 2. To inspect whether this condition also shows a decrease in tapping speed, the 10 s were split into 3 time bins.
