# Supplementary figures and images for: Finger tapping at maximal speed evokes crossover fatigability in the other hand
Source: Front Hum Neurosci. 2026 Apr 7;20:1782120. doi: 10.3389/fnhum.2026.1782120 (PMC13095690; doi:10.3389/fnhum.2026.1782120)

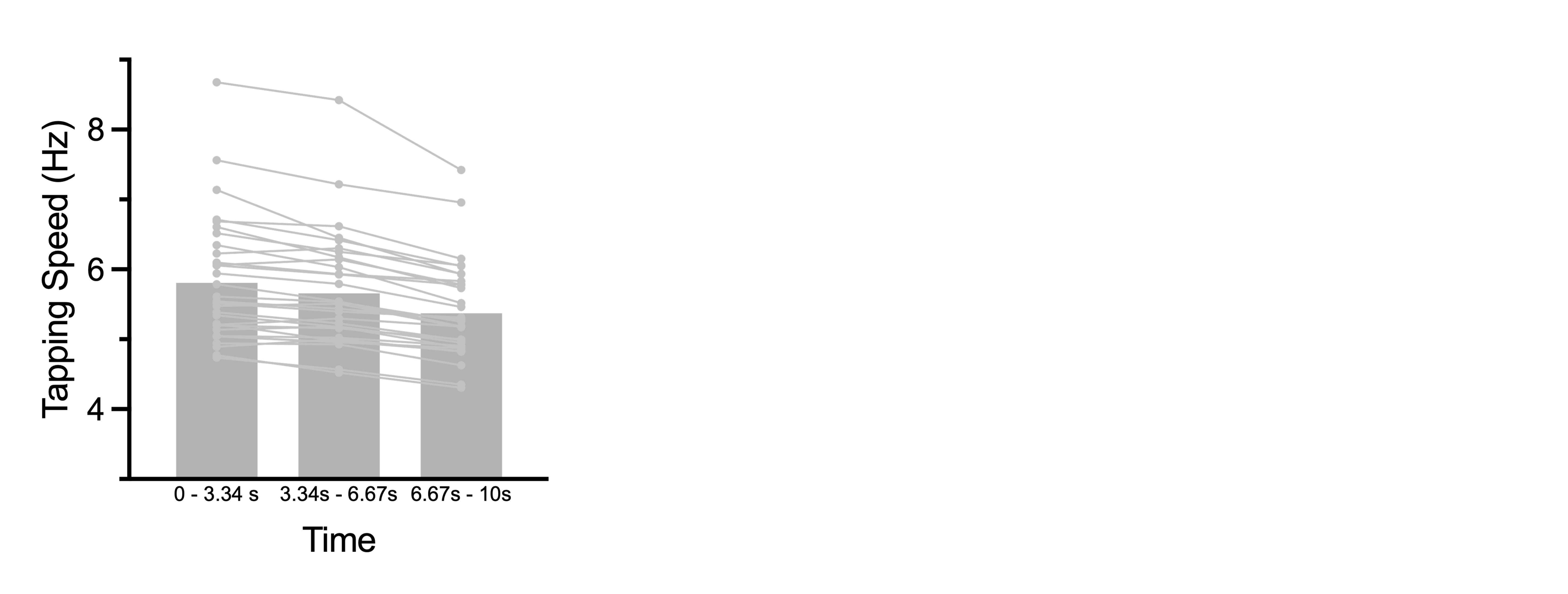

Supplement: Supplementary Figure 1 — 10 s tapping condition of the first hand of experiment 2. To inspect whether this condition also shows a decrease in tapping speed, the 10 s were split into 3 time bins. [file Image_1.tiff]
